# Feasibility of a twitter campaign to promote HPV vaccine uptake among racially/ethnically diverse young adult women living in public housing

**DOI:** 10.1186/s12889-020-08824-0

**Published:** 2020-06-01

**Authors:** Jennifer D. Allen, Justin Hollander, Lisa Gualtieri, Tania M. Alarcon Falconi, Stephanie Savir, Madina Agénor

**Affiliations:** 1 Department of Community Health, Tufts University, School of Arts and Sciences, Medford, MA USA; 2 Department of Urban and Environmental Policy and Planning, Tufts University, School of Arts and Sciences, Medford, MA USA; 3 Department of Public Health and Community Medicine, Tufts University School of Medicine, Boston, USA; 4 Tufts University, Friedman School of Nutrition Science and Policy, Boston, MA USA; 5 Department of Urban and Environmental Policy and Planning, Tufts University, School of Arts and Sciences, Medford, MA USA; 6 Department of Community Health, Tufts University, School of Arts and Sciences, Medford, MA USA

**Keywords:** Human papilloma virus, Vaccination, Health communication, Twitter, Low-income, Public housing

## Abstract

**Background:**

Uptake and completion of the HPV vaccine is suboptimal. This study assessed the feasibility of implementing a one-month Twitter campaign to promote knowledge about the human papillomavirus (HPV) vaccine among low-income women living in public housing.

**Methods:**

We recruited a convenience sample (*n* = 35) of women ages 18–26 years residing in low-come, public housing in Massachusetts. We assessed the feasibility and acceptability of a communication campaign that consisted of daily Twitter messages. Online surveys assessed changes in HPV knowledge, attitudes, and vaccine intentions before and after the campaign.

**Results:**

Most believed that Twitter was an acceptable educational strategy and remained engaged with the campaign throughout the intervention. We observed no changes in HPV knowledge, perceived benefits of or barriers to vaccination, decision self-efficacy, or vaccine intentions after the campaign, although perceived risk for cervical cancer decreased.

**Conclusions:**

Twitter may be a feasible and acceptable method for promoting knowledge about the HPV vaccine, but more research is needed to understand how best to reach low-income women with low levels of vaccine uptake.

**Trial registration:**

Clinicaltrials.gov 1,603,045, retrospectively registered 0610/19.

## Background

The American Cancer Society estimated that, in 2018, there were 13,240 new cases of invasive cervical cancer and 4170 cervical cancer deaths among women in the United States [1]. With the availability of prophylactic human papillomavirus (HPV) vaccines, it is possible to prevent the vast majority of cervical cancer cases. At the time of this study, medical organizations recommended that 3 doses of the vaccine be administered to girls ages 11–12 years, with “catch up” vaccination for women ages 13–26 years who had not previously received 3 doses [2].

Despite the availability of the HPV vaccine, initiation and completion of the series remains suboptimal. Results from the 2010 National Health Interview Survey (NHIS) revealed that less than 25% of U.S. women ages 21–30 years had initiated vaccination, and only 13% had completed the 3-dose series recommended at that time [3]. A more recent analysis of the 2015 NHIS among women ages 18–31 years found higher levels of vaccine initiation and completion (35 and 23%, respectively) [4], although uptake and completion is still well below national goals. National data show that while vaccine initiation does not differ by race/ethnicity, vaccine completion is significantly lower among black women relative to white women, although this association is somewhat attenuated after adjusting for socioeconomic factors [4]. Regardless, low levels of vaccine completion are concerning, especially given that low-income women and women of color experience a disproportionate burden of cervical cancer morbidity and mortality [5].

The use of social media to deliver public health campaigns is receiving increased attention [6]. Social media platforms such as Twitter, a micro-blogging platform, may be effective channels for the dissemination of public health interventions, especially given that 88% of those age 18–29 years report using a social networking site of some kind, and of these, 40% report using Twitter [7, 8]. There are numerous advantages to the use of Twitter for health communication campaigns, including the fact that it is free, can reach a wide, geographically dispersed audience, and can be used for short, text-based health messages. Additionally, campaign developers can obtain real-time feedback about the audience’s receptivity to health messages and subsequently re-craft them if initial reactions are not positive. Moreover, messages can be retweeted (or shared) among users, which offers the potential for greater dissemination and a broader impact. The delivery of online interventions also eliminates some of the known barriers to program participation, such as transportation and child care [8, 9].

A systematic review (*n* = 137) found that more than half (57%) of studies pertaining to Twitter and health involved content analyses of tweets to describe public discourse on a particular health topic. To a lesser extent, researchers have monitored and promoted online health communication on Twitter by assessing retweets and likes, for example. Few studies (14%) specifically tested the effect of implementing any kind of health campaign [8]. Several more recent studies have used Twitter to measure online communication specifically pertaining to HPV vaccination by assessing outcomes such as re-tweets, “likes,” and impressions [10–12]. However, we were only able to identify one intervention study of a Twitter campaign to promote HPV vaccine awareness, although numerous other studies have used other social media platforms [13]. In that study (*n* = 782) women were recruited online and completed surveys before and after a 5-day Twitter campaign about the HPV vaccine. They observed a statically significant increase in awareness of the HPV vaccine following the campaign (90 to 94%, *p* = 0.003), but no change in HPV vaccine uptake. Notably, 85% of participants had already received 3 doses of the vaccine at the beginning of the study. Nonetheless, Twitter analytics showed a high level of engagement with the campaign [13]. Given the limited information about Twitter-based HPV vaccine promotion interventions, particularly among women from lower socioeconomic backgrounds and women of color, the goal of this study was to assess the feasibility and preliminary effect of a month-long Twitter campaign among low-income and racially/ethicnally diverse group of women.

## Methods

The Health Belief Model (HBM) guided the study [14]. HBM theorizes that knowledge and perceptions about disease susceptibility and severity, perceived barriers and benefits, and self-efficacy ultimately drive health behaviors, and that intention to enact a behavior predicts behavioral action. According to the model, those with higher levels of perceived susceptibility and perceived benefits, as well as self-efficacy, are more likely to engage in health behaviors, as are those who perceive low barriers to the behavior.

### Sample and setting

We recruited a convenience sample of English-speaking women ages 18–26 years who were residents of public housing in two Massachusetts cities. We elected to recruit women from public housing in order to reach women with low levels of income; in the state of Massachusetts, those eligible to live in public housing must meet specific income requirements (i.e.,< 80% of the area median family income) [15]. We did not require that women have an existing Twitter account, but they had to be willing to do so for the study. We also did not restrict eligibility to those who had not previously received the vaccine, as we were interested in assessing the feasibility of offering the Twitter campaign and in addition, wanted to assess cervical cancer screening practices in this high-risk population. Our goal was to recruit 50 women over a two-month period. To recruit women, we mailed information about the study, posted fliers in common use areas in housing developments, and with the housing authorities, transmitted email or phone announcements about the study. Women were informed that this was a a research study on “women’s health.”

### Intervention

Women received a daily tweet over a period of one-month that contained messages from educational materials produced by the Centers for Disease Control and Prevention, National Cancer Institute and the Massachusetts Department of Public Health. Messages were selected to align with the Health Belief Model and primarily addressed HPV vaccination, although there were also messages promoting cervical cancer screening (see Table 1).

**Table 1:** Sample campaign messages by HBM constructs

Construct	Tweet
Perceived risk	-You can get HPV by having oral, vaginal, or anal sex with someone who has the virus. HPV can be passed even when an infected person has no signs or symptoms. -Nearly 80 million people in the US are currently infected with HPV. Most sexually-active men & women will get some type of HPV at some point in their lives. -Sexually active? You may contract HPV infection. Don’t risk it, get the vaccine.
Perceived benefits	-The HPV vaccine protects the reproductive health of girls and women. Know the facts! -Most HPV infections go away without treatment, but some types of HPV can cause cancer, including cervical cancer. Don’t risk it, get the vaccine. -No woman should die from cervical cancer. It is preventable with the HPV vaccine and detected early with a test called the Pap test. -HPV vaccines work extremely well. HPV vaccines provide close to 100% protection against cervical precancers and genital warts.
Perceived barriers	-One simple conversation could save your life. Talk with your doctor about the HPV vaccine. -Many people who get the HPV vaccine have no side effects at all. Some people report having very mild side effects, like a sore arm.

### Data collection

Pre/post surveys were administered by phone or online and assessed cervical cancer screening, HPV and HPV vaccine knowledge, perceived risk for cervical cancer, perceived barriers to HPV vaccination, and HPV vaccination intentions. Upon completion of the campaign, we contacted women through Twitter to let them know that we would be calling them to complete the post-survey. Women received a $50 gift card for participation. All procedures and protocols were approved by the Institutional Review Board at Tufts University.

### Assessment of feasibility

Feasibility was assessed with standard metrics, for example, by determining the numbers of women who: were recruited relative to recruitment goals, had Twitter accounts at the time of recruitment, continued to receive our messages over the one-month period (i.e., did not block messages), and who completed the post-test survey. Twitter analytics were used to track re-tweets and “likes.” To assess receipt and recall of messages, participants were asked at the end of the post-test to indicate whether they received daily tweets from the campaign. The first question was: *“Did you receive Twitter messages about HPV from the Tufts Women’s Health Study?”* (yes/no). Next, we asked: *“Which of the following messages did you receive from the Tufts Women’s Health Study?”* Response options included true statements that were emphases of the campaign (e.g., “HPV vaccine can prevent cervical cancer”) and also false statements not included in the campaign (e.g., “Most women will feel symptoms if they have the HPV infection, so it is often detected early”). In addition, women were asked about the acceptability of receiving Twitter messages at the end of the study.

### Measures of health belief model constructs

When standardized questions were not available, we adapted items from our prior studies, which had high reliability [16]. Items to assess receipt of the HPV vaccine, Pap testing, and sociodemographic characteristics were taken from the BRFSS [17]. Intention to be vaccinated among those who had not completed the 3-dose series was assessed by asking “How likely is it that you will get vaccinated against HPV in the future? In the next 6 months? In the next 12 months?” with responses on a 4-point Likert scale from very likely to very unlikely. Additional survey items assessed usual source of care, health insurance, and selected sociodemographic based on standard items from national surveys [18].

*HPV and cervical cancer knowledge* was assessed with 13 items that addressed knowledge about HPV, the HPV vaccine, and cervical cancer; HPV and cervical cancer risk factors and HPV transmission [16]. For each correct answer, respondents received one point; for each “don’t know” or incorrect answer, respondents received no point. Total points were then divided by the maximum number of points and multiplied by 100 to arrive at a scale with a range of 0–100%.

*Perceived susceptibility to HPV* and *perceived susceptibility to cervical cancer* were each assessed with 2 items that addressed overall perceived risk (e.g., *“Overall, how would you rate your chance of developing HPV?”*) and risk compared to similar-aged peers (e.g., *“How would you rate your chances of developing cervical cancer compared to average women your age?”)* [19]. Response options were on a 5-point Likert scale (ranging from 1 = “very low” to 5 = “very high”). “Don’t know” responses were coded as 0. Points were summed up for the 2 items in each score such that higher values reflect higher perceived risk (range 0–10).

*Perceived benefits of HPV vaccination* were assessed with two composite scores: vaccine efficacy and vaccine safety [16]. *Vaccine efficacy* was assessed with 3 items that examined the potential for vaccination to prevent HPV infection, genital warts, and cervical cancer (e.g., “*In your opinion, how effective is the HPV vaccine in preventing HPV infection?”*). Response options were on a 4-point Likert scale (ranging from 1 = “not at all effective” to 4 = “very effective”). “Don’t know” responses were coded as 0. Points were summed up for the 3 items such that higher values reflect greater perceived efficacy (range 0–12). V*accine safety* was assessed with two items that addressed safety and likelihood of the vaccine causing other health problems. Safety response options were on a 4-point Likert scale (ranging from 1 = “not at all safe” to 4 = “very safe”), while likelihood of other health problems was on a 5-point Likert scale (ranging from 1 = “very likely” to 5 = “never”). “Don’t know” responses were coded as 0. Points were summed up for the 2 items such that higher values reflect greater perceived safety (range 0–9).

*Perceived barriers to vaccination* included assessment of potential pain and cost associated with vaccination with 2 items: *“In your opinion, how painful [expensive] would it be to receive the HPV vaccine?”* with response options on a 4-point Likert scale (ranging from 1 = “not at all painful [expensive]” to 4 = “very painful [expensive]”) [20]. “Don’t know” responses were coded as 0. Responses were combined such that higher scores reflect greater perceived barriers (range 0–8).

*Decision self-efficacy* with regard to obtaining and comprehending information about the vaccine was assessed with 11 items adapted from the Decision Self-Efficacy Scale [21] (e.g., "*How confident do you feel to get the facts about the risks of the HPV vaccine?*). Responses were on a 2-point Likert scale (ranging from 0 = “not at all confident” to 2 = “very confident”). Total points were then divided by the maximum number of points and multiplied by 100 to arrive at a scale with a range of 0–100%, with higher scores indicating higher confidence.

### Analysis

Data from pre- and post-test surveys were analyzed using R and RStudio [22, 23]. Descriptive statistics, including means and standard deviations (SD), were used to describe the sample in terms of sociodemographic characteristics and HPV vaccination knowledge, attitudes, and behaviors. Assessment of feasibility involved descriptive data (e.g., number of women who recalled receiving messages) as well as examination of Twitter analytics (e.g., likes, retweets). Changes between pre- and post-test were analyzed with paired t-tests for continuous variables (i.e., knowledge, perceived susceptibility, perceived benefits of and barriers to HPV vaccination, and decision self-efficacy) and McNemar’s test for categorical variables (i.e., intention to get the HPV vaccine in the future among those who had not completed vaccination).

## Results

### Assessment of feasibility

Of the 42 women initially recruited, 35 (83%) also completed the post-test survey and 7 initiated, but did not complete the post-test survey. At the time of enrollment, approximately half (57%) had an existing Twitter account. About a third (36%) of those with an existing Twitter account said they checked on a daily basis, although a higher percentage (45%) said they checked every few weeks or less. During the campaign, six of the women “liked” our Tweets and four of the women “retweeted” our messages (likes = 55, retweets 84 times). Of the 35 women who completed the study, only 2 (5.7%) ‘blocked’ our tweets during the campaign. At post-test, 71% of women agreed with the statement “*Twitter messages are a good way to educate women about the HPV vaccine*.”

### Participant characteristics (Table 2)

Participants who completed both surveys ranged in age from 18 to 26 years (mean 21.9, SD = 2.6). About two-thirds (63%) had household incomes below $50,000/year, two-thirds (61%) had at least some college education, half (54%) had public insurance, and the majority (91%) were single. Half (51%) were black or African American, and 40% were born outside of the U.S. from a wide variety of countries with highest percentage from Caribbean (20%). Less than half (40%) had received a Pap test in the last 3 years. The majority (94%) was aware of HPV infection, and 63% had received 3 doses of the HPV vaccine.

**Table 2:** Participant Sociodemographic Characteristics (n = 35)

**Age in years (mean, SD)**	21.89 (2.63)
	**n (%)**
**Race/ethnicity**	
Non-Hispanic white	5 (14)
Non-Hispanic black	18 (51)
Hispanic	0 (0)
More than one race	3 (9)
Another race or ethnicity	7 (20)
**Household Income**	
≤ $25,000	11 (31)
$25,001–$50,000	11 (31)
$50,001–$75,000	8 (23)
> $75,000	3 (9)
**Educational Level**	
< High school	3 (9)
High school graduate/GED	8 (23)
Some college/2-year degree	16 (46)
4-year college degree	7 (20)
> 4-year college degree	1 (3)
**Health insurance Status**	
Public	19 (54)
Private	12 (34)
Combination public & private	3 (9)
None	1 (3)
**Usual source of care**	
Yes	34 (97)
No	1 (3)
**Marital Status**	
Married/living as married	3 (9)
Single/never married	32 (91)
**Received Pap test in last 3 years**	
Yes	14 (40)
No	19 (54)
Don’t know	2 (6)
**HPV Vaccination Status**	
Fully vaccinated (3 doses)	22 (63)
Initiated vaccination (at least 1 dose)	1 (3)
Don’t know	6 (17)

Note. Percentages may not total 100% due to rounding or missing data

### Health Belief Model constructs (Table 3)

Before the campaign, HPV knowledge scores were low, and we observed no statistically significant change after the campaign (56% vs 57%, *p* = 0.858). Perceived risk of developing cervical cancer was low and decreased at post-test (4.46 vs. 3.37 out of 10; *p* = 0.018), although this change was no longer statistically significant in subgroup analyses among women who verified receipt of messages. Participants generally perceived that there were high benefits to HPV vaccination in terms of vaccine efficacy (mean of 9 on a scale of 0–12) and vaccine safety (mean of 6 on a scale of 0–9). They tended to report that there were only moderate barriers to HPV vaccine uptake (mean of 4 on a scale of 1–8). HPV vaccination decision self-efficacy was high and did not change after the campaign (85% vs 83%, respectively, *p* = 0.95). We observed no statistically significant change in the intent to be vaccinated in the next 6-months (*p* = 1.000) or 12-months (*p* = 0.617) after the campaign among those who had not yet started or completed vaccination.

**Table 3:** Changes in Health Belief Model Constructs between Pre- and Post-Test (n = 35)

	Pre-test (N = 35)	Post-test (N = 35)	
	**mean (SD)**	**mean (SD)**	***p*-value**^**a**^
**HPV & cervical cancer knowledge (0–100%)**	56.30 (18.95)	56.96 (19.33)	0.858
**Perceived susceptibility**			
Cervical cancer (0–10)	**4.46 (1.70)**	**3.37 (2.10)**	**0.018**
HPV infection (0–10)	3.43 (1.46)	3.03 (1.69)	0.295
**Perceived benefits of HPV vaccination**			
HPV vaccine efficacy (0–12)	9.26 (3.20)	9.00 (3.64)	0.638
HPV vaccine safety (0–9)	6.14 (1.75)	5.63 (2.17)	0.074
**Perceived barriers to vaccination (0–8)**	4.37 (1.59)	3.89 (1.86)	0.195
**Decision self-efficacy (0–100%)**	84.71 (13.76)	82.90 (19.12)	0.955
	**N (%)**	**N (%)**	**p-value**^**b**^
**Intention to obtain vaccine among those with < 3 doses (n = 7)** Very likely/likely in next 12 months	5 (71)	4 (57)	1.000
Very likely/likely in next 6 months	5 (71)	3 (43)	0.617

Note. Statistically significant differences are bolded

^a^ Based on paired t-test

^b^ Based on McNemar’s test

## Discussion

Twitter is a relatively new innovation and its utility as a mode of delivering health campaigns has not been fully explored. We aimed to assess the feasibility of a Twitter campaign on HPV vaccination among low-income women from diverse racial/ethnically diverse young adult women recruited from public housing. Given the dearth of information on the feasibility and impact of social media interventions on health beliefs, attitudes, and behaviors among socially and economically marginalized populations who experience health and health information disparities [8], our findings suggest that it is feasible use Twitter to disseminate HPV-related messages to this audience- -even among those who did not have an existing Twitter account. Indeed, the majority of women remained in the study for the intervention period and few blocked our health promotion messages. Furthermore, most participants found Twitter to be an acceptable means of receiving HPV vaccine information, as measured by post-test surveys.

Before discussing study implications, we note limitations. First, we focused on feasibility and recruited a small, non-probability sample of young adult women. As a result, findings may not be generalizable to other populations and it is unlikely that there was adequate statistical power to detect any intervention effect. Our study also did not include a control group to whom findings among the intervention group could be compared. Therefore, we were not able to determine whether any change observed in the intervention group was due to the intervention itself or other factors. Lastly, there has been concern that social media-based interventions could exacerbate health disparities due to differential access to the Internet and smart phones among social groups. However, we elected to use Twitter because the majority of young adults living in urban areas in the U.S. use this social media platform [24]. Future research that relies on a larger probability sample, includes a control group, and assesses any unintended consequence on health disparities is needed.

It is possible that we did not observe any effect of the intervention on HPV vaccination because study participants (self-selected) had high vaccine completion at baseline. It is possible that this high uptake is a result of good access to healthcare in Massachusetts. Massachusetts passed healthcare reform in 2006, with the goal of providing health insurance to most residents. The law mandates that Massachusetts provide free or subsidized healthcare insurance for residents earning less than 150 and 300% of federal poverty level. As a result, residents of Massachusetts have greater access to preventive health care compared with other states.

Alternatively, it could be that Twitter users are more willing to get the vaccine. The only other Twitter-based HPV vaccination intervention study that we identified, which also did not find any effect of the intervention on HPV vaccine uptake among women, also observed high levels (85%) of HPV vaccine completion at baseline [13]. Thus, additional research is needed to assess whether Twitter-based interventions can truly reach women with low levels of HPV vaccine uptake. Studies suggest that different strategies may be needed to effectively reach women low levels of vaccine adoption.

Notably, the use of Twitter in our sample was lower than for the general population in this age group (22%). Twitter use is generally higher among those under age 50 years and those living in urban rather than rural areas [24], which led us to explore this platform in the current study. Nationally, Twitter users are generally more highly educated and have higher incomes compared with the general U.S. population [25]. Along gender and racial dimensions, however Twitter users are quite representative of the general population. Data from Pew Research Center found that, in 2018, more young adults used Instagram and Snapchat than Twitter [26]. It may well be that using these other social media platforms could better reach our intended audience, specifically those with low vaccine uptake. However, even with heavier adoption of other platforms, Twitter may have advantages over Instagram and Snapchat since those are more graphical and text-based messages, though scholars have debated the pros and cons of each [27–29].

In addition to considering alternative social media platforms, it is unlikely that dissemination of information alone will change HPV vaccine behaviors. The Community Guide recommends that cervical cancer interventions utilize multiple components, such as group education [30]. Women in this study did not have any opportunity to interact with each other and messaging was unidirectional. Adding a social connection or support component may enhance the impact of this and other social media interventions [31, 32].

## Conclusions

More research is needed to assess the messages, dose, and social media platform that most effectively facilitate HPV vaccine knowledge and uptake in socially and economically marginalized populations at risk of cervical cancer [33, 34]. Moreover, studies conducted among women with low levels of HPV vaccine awareness and uptake are also warranted. Lastly, additional studies are needed to understand the extent to which the dissemination of HPV vaccine promotion messages through Twitter can affect HPV-related outcomes among geographically dispersed groups beyond the intervention group. Together, these efforts may help facilitate the dissemination of information and possibly affect HPV vaccine knowledge, risk perceptions, and vaccination behaviors among marginalized populations at high risk of developing cervical cancer.

## Data Availability

The dataset generated and analyzed during the current study are not publicly available but are available from the corresponding author on reasonable request.

## Abbreviation

HPV: Human papilloma virus

## References

[1] Street - 1930 - Cancer Facts & Figures 2018.pdf. https://www.cancer.org/content/dam/cancer-org/research/cancer-facts-and-statistics/annual-cancer-facts-and-figures/2018/cancer-facts-and-figures-2018.pdf. Accessed 21 Mar 2019.

[2] Meites E, Kempe A, Markowitz LE (2017). Use of a 2-dose schedule for human papillomavirus vaccination—updated recommendations of the advisory committee on immunization practices. Am J Transplant.

[3] Williams WW, Lu P-J, O’Halloran A (2017). Surveillance of Vaccination Coverage among Adult Populations — United States, 2015. MMWR Surveill Summ.

[4] Agénor M, Pérez AE, Peitzmeier SM, Borrero S. Racial/ethnic disparities in human papillomavirus vaccination initiation and completion among U.S. women in the post-Affordable Care Act era. Ethn Health. 2018:1–15. 10.1080/13557858.2018.1427703. 10.1080/13557858.2018.1427703 29347831

[5] USCS Data Visualizations. https://gis.cdc.gov/grasp/USCS/DataViz.html. Accessed 21 Mar 2019.

[6] Korda H, Itani Z (2013). Harnessing social media for health promotion and behavior change. Health Promot Pract.

[7] Gough A, Hunter RF, Ajao O, et al. Tweet for Behavior Change: Using Social Media for the Dissemination of Public Health Messages. JMIR Public Health Surveill. 2017;3(1). doi:10.2196/publichealth.6313. 10.2196/publichealth.6313 PMC5383801 28336503

[8] Sinnenberg L, Buttenheim AM, Padrez K, Mancheno C, Ungar L, Merchant RM (2017). Twitter as a tool for Health Research: a systematic review. Am J Public Health.

[9] Shi J, Poorisat T, Salmon CT (2018). The Use of social networking sites (SNSs) in health communication campaigns: review and recommendations. Health Commun.

[10] Lenoir P, Moulahi B, Azé J, Bringay S, Mercier G, Carbonnel F (2017). Raising awareness about cervical Cancer using twitter: content analysis of the 2015 #SmearForSmear campaign. J Med Internet Res.

[11] Teoh D, Shaikh R, Vogel RI (2018). A cross-sectional review of cervical Cancer messages on twitter during cervical Cancer awareness month. J Low Genit Tract Dis.

[12] Xu S, Markson C, Costello KL, Xing CY, Demissie K, Llanos AA (2016). Leveraging social media to promote public health knowledge: example of Cancer awareness via twitter. JMIR Public Health Surveill.

[13] Ortiz RR, Shafer A, Cates J, Coyne-Beasley T. Development and Evaluation of a Social Media Health Intervention to Improve Adolescents’ Knowledge About and Vaccination Against the Human Papillomavirus. Glob Pediatr Health. 2018;5:2333794X18777918. doi:10.1177/2333794X18777918. 10.1177/2333794X18777918 PMC5977424 29872667

[14] The Health Belief Model and Preventive Health Behavior - Irwin M. Rosenstock, 1974. https://journals.sagepub.com/doi/abs/10.1177/109019817400200405?journalCode=heba. Accessed 21 Mar 2019.

[15] PHA Contact Information - HUD | HUD.gov / U.S. Department of Housing and Urban Development (HUD). https://www.hud.gov/program_offices/public_indian_housing/pha/contacts. Accessed 21 Mar 2019.

[16] Allen JD, Mohllajee AP, Shelton RC, Othus MKD, Fontenot HB, Hanna R (2009). Stage of adoption of the human papillomavirus vaccine among college women. Prev Med.

[17] CDC - 2016 BRFSS survey data and documentation. https://wwwcdcgov/brfss/annual_data/annual_2016html Published February 20, 2019. Accessed 21 Mar 2019.

[18] NHIS - National Health Interview Survey Homepage. https://www.cdc.gov/nchs/nhis/index.htm. Published March 20, 2019. Accessed 21 Mar 2019.

[19] Weinstein ND, Kwitel A, McCaul KD, Magnan RE, Gerrard M, Gibbons FX (2007). Risk perceptions: assessment and relationship to influenza vaccination. Health Psychol.

[20] Allen JD, Othus MKD, Shelton RC (2010). Parental decision making about the HPV vaccine. Cancer Epidemiol Biomark Prev Publ Am Assoc Cancer Res Cosponsored Am Soc Prev Oncol.

[21] O’Connor AM (1995). Validation of a decisional conflict scale. Med Decis Mak Int J Soc Med Decis Mak.

[22] Citing RStudio. RStudio Support. http://support.rstudio.com/hc/en-us/articles/206212048-Citing-RStudio. Accessed 21 Mar 2019.

[23] R: a language and environment for statistical computing. https://www.gbif.org/tool/81287/r-a-language-and-environment-for-statistical-computing. Accessed 21 Mar 2019.

[24] Demographics of Social Media Users in 2015. August 2015. https://www.pewinternet.org/2015/08/19/the-demographics-of-social-media-users/. Accessed 21 Mar 2019.

[25] Wojcik S, Hughes A. Sizing Up Twitter Users. Pew Research Center; 2019. https://www.pewresearch.org/internet/wp-content/uploads/sites/9/2019/04/twitter_opinions_4_18_final_clean.pdf. Accessed 9 Mar 2019.

[26] Social Media Use 2018: Demographics and statistics | pew research center. March 2018. https://www.pewinternet.org/2018/03/01/social-media-use-in-2018/. Accessed 21 Mar 2019.

[27] Pittman M, Reich B (2016). Social media and loneliness: why an Instagram picture may be worth more than a thousand twitter words. Comput Hum Behav.

[28] Guidry JPD, Jin Y, Orr CA, Messner M, Meganck S (2017). Ebola on Instagram and twitter: how health organizations address the health crisis in their social media engagement. Public Relat Rev.

[29] Hollander JB, Graves E, Renski H, Foster-Karim C, Wiley A, Das D. Urban social listening: Potential and pitfalls for using microblogging data in studying cities. Palgrave Macmillan; 1st ed. 2016. 10.1057/978-137-49491-4.

[30] Cancer Screening: Multicomponent Interventions—Cervical Cancer. The Guide to Community Preventive Services (The Community Guide). https://www.thecommunityguide.org/findings/cancer-screening-multicomponent-interventions-cervical-cancer. Published February 9, 2017. Accessed 21 Mar 2019.

[31] Heldman AB, Schindelar J, Weaver JB. Social Media Engagement and Public Health Communication: Implications for Public Health Organizations Being Truly “Social.” Public Health Rev 2013;35(1):13. doi:10.1007/BF03391698.

[32] Zhou X, Coiera E, Tsafnat G, Arachi D, Ong MS, Dunn AG. Using social connection information to improve opinion mining: Identifying negative sentiment about HPV vaccines on Twitter. In: Studies in Health Technology and Informatics. Vol 216. IOS Press; 2015:761–765. doi:10.3233/978-1-61499-564-7-761. 26262154

[33] Paek H-J, Oh S-H, Hove T (2016). How fear-arousing news messages affect risk perceptions and intention to talk about risk. Health Commun.

[34] Witte K, Allen M (2000). A meta-analysis of fear appeals: implications for effective public health campaigns. Health Educ Behav Off Publ Soc Public Health Educ.

